# Psychosis in muslim arab population. Case report and article review

**DOI:** 10.1192/j.eurpsy.2021.866

**Published:** 2021-08-13

**Authors:** R. Sagarra Arruego, E. Ramos García, F.L. Bianchi Ramos, Á. Martínez Fernández, R. Molina Cambra, A. Muñoz Domenjó, M. Ortega Moreno, M. Hernández Barrera

**Affiliations:** Psychiatry, University Hospital of Móstoles, Madrid, Spain

**Keywords:** psychosis, arab, muslim, transcultural psychiatry.

## Abstract

**Introduction:**

In Spain, we are forced to familiarize ourselves with Arab-Muslim culture to properly treat our patients. The diagnosis becomes complicatedbecause western health professionals are not usually familiar with thisform of symptom presentation.

**Objectives:**

The objective of this work is to study the influence of Arab culture and Muslim religion on the psychopathological symptoms presented duringa psychotic episode.

**Methods:**

We present two cases of psychosis in two brothers of Maghreb originwho were treated for the first psychotic episode in the acute psychiatricunit in a Spanish regional hospital. Then, we carried out a litle researchfrom the literatura.

**Results:**

The common psychopathological symptoms presented by two brothersof 26 and 27 years were: symptoms of thought, control and influence of the self. Delusional ideas of self-referential harm and persecution. Auditory and cenesthetic hallucinations. In the literature we find that patients with Islamic backgrounds whosuffer hallucinations can attribute these experiences to different beliefssuch as geniuses (jinn), black magic and the evil eye. One of the siblings was diagnosed with a psychotic episode withoutspecification, while the other brother got the schizophrenia label. Webelieve that this may be related to the fact that mental healthprofessionals generally tend to label fantastic stories as mind-blowingor delusional in nature.

**Conclusions:**

1. Religious beliefs and fantastic tales of Muslim culture can be considered psychotic symptoms if healthcare professionals are notfamiliar with this culture. 2. Teamwork between mental health professionals, translators and religious counselors can improve care for Muslim patients.

